# Post-Traumatic Left Subclavian Artery Pseudoaneurysm Secondary to Clavicular Fracture: A Case Report and Literature Review

**DOI:** 10.3390/biomedicines13010187

**Published:** 2025-01-14

**Authors:** Małgorzata Edyta Wojtyś, Patryk Skórka, Dawid Kordykiewicz, Aleksander Falkowski, Joanna Jakubowska-Grzeszyk, Janusz Wójcik, Edward Michael Wojtys

**Affiliations:** 1 Department of Thoracic Surgery and Transplantation, Pomeranian Medical University in Szczecin, Alfreda Sokołowskiego 11, 70-891 Szczecin, Poland; 2 Department of General, Dental and Interventional Radiology, Pomeranian Medical University in Szczecin, Powstańców Wielkopolskich 72, 70-111 Szczecin, Poland; 3 Department of Radiology, SPWSZ in Szczecin, Sokołowskiego 11, 70-891 Szczecin, Poland; 4 Department of Orthopaedic Surgery, University of Michigan, Ann Arbor, MI 48109, USA

**Keywords:** pseudoaneurysm, subclavian artery, endovascular treatment, clavicular fracture

## Abstract

Subclavian artery pseudoaneurysms are rare but potentially life-threatening vascular injuries frequently associated with trauma such as clavicle fractures. In this paper we describe the case of a 49-year-old male who developed a post-traumatic pseudoaneurysm of the subclavian artery after a bicycle accident. The diagnosis was delayed due to non-specific symptoms and an initially missed aneurysm on computed tomography imaging. Persistent pain, swelling, and erythema in the subclavian region prompted further detailed diagnostics, which ultimately revealed the pseudoaneurysm. The patient was successfully treated with endovascular stent–graft implantation. We screened the PubMed database to identify similar cases managed exclusively through endovascular intervention. Reports of iatrogenic pseudoaneurysms and those treated with open surgery were excluded. Variables such as time to diagnosis, clinical presentation, features of pseudoaneurysms, and complications were analyzed to highlight the role of endovascular techniques as a minimally invasive and effective treatment option. These cases pose both a diagnostic and a therapeutic challenge, as early recognition of symptoms is crucial to prevent serious complications including thrombosis, neurological deficits, and even limb loss.

## 1. Introduction

Arterial injuries, like those affecting other structures, can occur in isolation or as part of multisite or multi-organ trauma. Depending on the mechanism of injury, they can be classified as direct or indirect. Due to the quite frequent course of large arteries in the vicinity of bones, these injuries are often associated with trauma, usually osteochondral fractures. Excluding iatrogenic injuries, which are not the object of this article, arterial injuries can be divided between open injuries (cuts, marginal injuries, or rupture) and closed injuries such as contusion, crushing, or intravascular damage to one or more layers of the vessel wall [1].

The diagnosis of open arterial injury is usually not difficult. Symptoms of acute ischemia of the area supplied by the artery, arterial hemorrhage, or a progressive hematoma resulting from vessel damage are usual, although not always present. However, closed arterial injuries can be more challenging due to incomplete blood flow through the injured vessel and the presence of non-specific symptoms, which may delay diagnosis [2].

The subclavian artery (SCA) originates on the right side from the brachiocephalic trunk, and on the left from the aortic arch. It follows an upward convex course over the pleural pericardium, then passes laterally between the anterior and middle scalene muscles, running through the subclavian groove on the first rib. After crossing beneath the clavicle, the SCA continues as the axillary artery, which subsequently transitions into the brachial artery [3].

Fractures of the clavicle are commonly caused by falls from height or during recreational activities such as cycling, rollerblading, or scooter riding. The mechanism of clavicle fracture can be indirect trauma, such as a fall on the shoulder, or direct trauma, impact, or the force exerted by a seatbelt during a car accident. In indirect trauma, the clavicle usually breaks along its midpoint. More than 80% of clavicle fractures are shaft fractures. Damage to surrounding structures such as the SCA, subclavian vein, or brachial plexus is relatively rare [4].

Trauma-related SCA injury is a very rare complication occurring in <0.1% of trauma admissions patients, and among those treated for post-traumatic pseudoaneurysm it constitutes approximately 8% of all cases [5,6]. In around 50% of SCA injuries, a fracture of the clavicle and ribs accompanies the injury. Pseudoaneurysm occurs as a result of rupture of one or more layers of the arterial wall, and if the injury site does not close, arterial bleeding into the adjacent soft tissues follows, resulting in the formation of a hematoma. In some cases, a fluid area develops in the central part of the lesion, while the outer part of the lesion forms a hard pseudocapsule. This allows blood to flow outside the damaged vessel, ultimately leading to pseudoaneurysm formation. This pseudoaneurysm consists only of fibrous tissue without any muscular elements [7], characterized by localized defects in arterial integrity. It manifests as a pulsatile hematoma that maintains communication with the artery through a lesion in the arterial wall. These vascular anomalies are invariably associated with a significant risk of mortality. Pseudoaneurysm may be the result of various causes of injury such as gunshot, stab wound, bomb blast, or a failed attempt at central venous catheter placement [6,8,9,10]. However, such causes usually involve locations other than the subclavian. In the case of pseudoaneurysm of the SCA, the most common cause seems to be car accidents, and the direct cause is a fracture of the clavicle. Currently, there are no strict guidelines for the treatment of pseudoaneurysms of the SCA [11]. Pseudoaneurysm of the SCA can be treated with traditional open surgery or a minimally invasive endovascular procedure. Prompt diagnosis and intervention are critical for successful management. Delays in treatment can result in life-threatening complications, such as thromboembolism, neurological deficits, and even limb amputation.

The aim of this report is to present a case of endovascular treatment of a pseudoaneurysm in a patient following a bicycle accident, where initial lesions did not suggest vascular damage. In addition, we review similar case reports of endovascularly treated cases. Diagnostic difficulties and different patient strategies are described. We also address the clinical dilemma of managing a clavicle fracture in a patient with a post-traumatic left SCA pseudoaneurysm, highlighting the need for a careful and individualized treatment approach. This case underscores the importance of considering rare complications, such as pseudoaneurysms, following clavicle injuries, as they pose a significant health risk if overlooked.

## 2. Case Presentation

**Figure 1:**
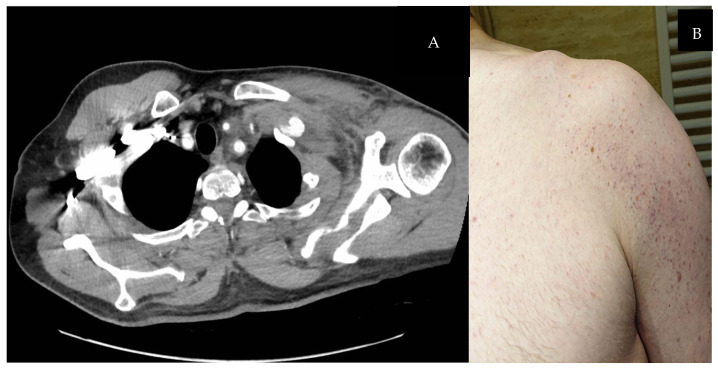
(**A**) Chest CT scan (axial plane): pseudoaneurysm misinterpreted as a massive hematoma of the supraclavicular region. (**B**) Patient’s subclavian region and shoulder during admitting.

A 49-year-old right-handed male was admitted to the thoracic surgery department following a bicycle accident, presenting with palpation-painful swelling of the left supraclavicular region, restricted mobility of the left glenohumeral joint, and left-sided chest pain. Upon arrival at the emergency department, a computed tomography (CT) scan of the entire body excluded intracranial abnormalities and cranial fractures. However, a massive hematoma in the supraclavicular region was described, but not initially recognized as a pseudoaneurysm (Figure 1).

**Figure 2:**
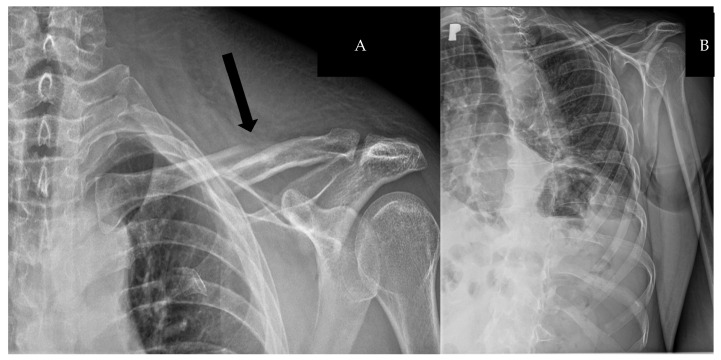
(**A**) Chest X-ray: oblique fracture of the clavicle shaft (black arrow). (**B**) Chest X-ray: flail chest with dual fractures of the second through seventh ribs on the left side.

An oblique fracture of the clavicle shaft and flail chest with fracture of the second through seventh ribs on the left side, located along the anterior axillary line and parasternal lines, was identified (Figure 2).

**Figure 3:**
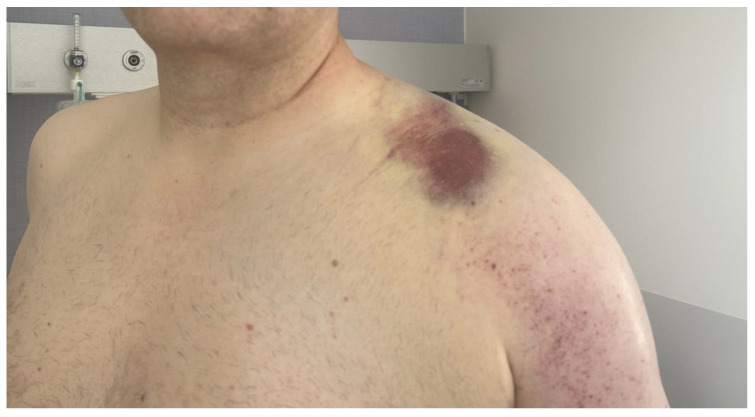
Swelling of the left supraclavicular region.

The patient qualified for conservative treatment in the department. Nevertheless, 10 days after admission, the swelling of the left supraclavicular region did not resolve (Figure 3).

**Figure 4:**
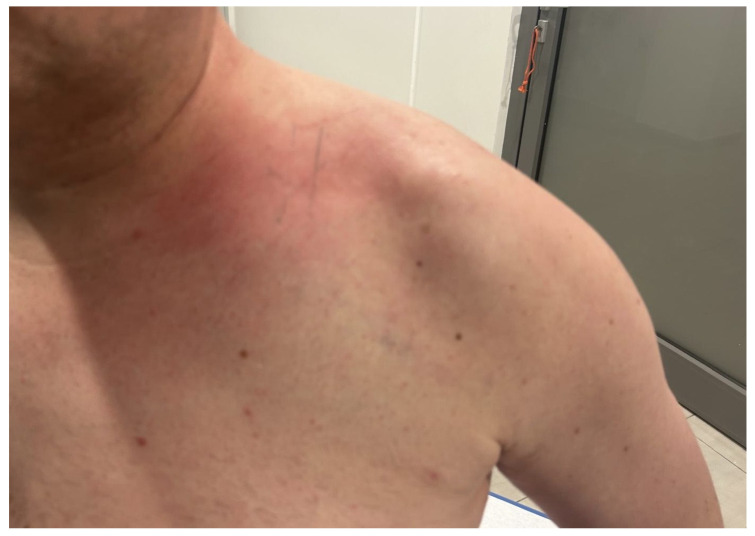
Clavicle and shoulder region at 7-week follow-up.

Additional swelling of the left upper limb prompted a Doppler ultrasonography, revealing thrombotic changes in the subclavian vein and brachial vein, along with a hematoma in the subclavian region. Laboratory results revealed elevated D-dimer levels at 2168.0 μg/L and a CRP level of 3.43 mg/L, indicative of subclavian and peripheral vein thrombosis. After consultation with a vascular surgeon, anticoagulant treatment including subcutaneous injections of low-molecular-weight heparin (LMWH) in therapeutic doses was administered. Following the treatment, the swelling resolved and partial recanalization of the subclavian vein was achieved. The patient was discharged home in good condition on rivaroxaban treatment for 3 months with a follow-up visit scheduled in 3 weeks. At the 3-week follow-up, the patient complained of persistent moderate swelling of the left supraclavicular region. Imaging examinations with ultrasound of the pleura, chest, and mediastinum, and chest X-ray and Doppler ultrasonography of the left upper limb, showed no abnormalities. One month later, during another outpatient visit, the symptoms persisted while laboratory and imaging studies showed no abnormalities (Figure 4). CRP and D-Dimer levels were 0.77 mg/L and 447.0 μg/L, respectively.

**Figure 5:**
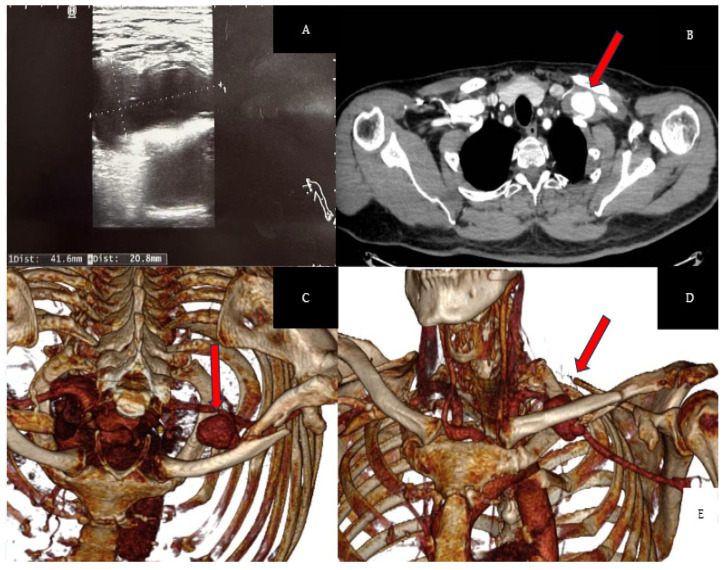
(**A**) Doppler ultrasonography indicated pseudoaneurysm. (**B**) Chest CT scan (axial plane) confirmed subclavian pseudoaneurysm. (**C**,**D**) 3-dimensional image showing the pseudoaneurysm (red arrows).

Four months post-injury, the patient presented with recurrent swelling in the left supraclavicular region and left-sided chest pain. Subsequent Doppler ultrasonography showed preserved flow in the subclavian vessel, but a partially thrombosed vessel measuring 40 × 20 mm was identified, suggesting a pseudoaneurysm. Angio-CT confirmed a 40 × 24 mm pseudoaneurysm of the left SCA (Figure 5).

**Figure 6:**
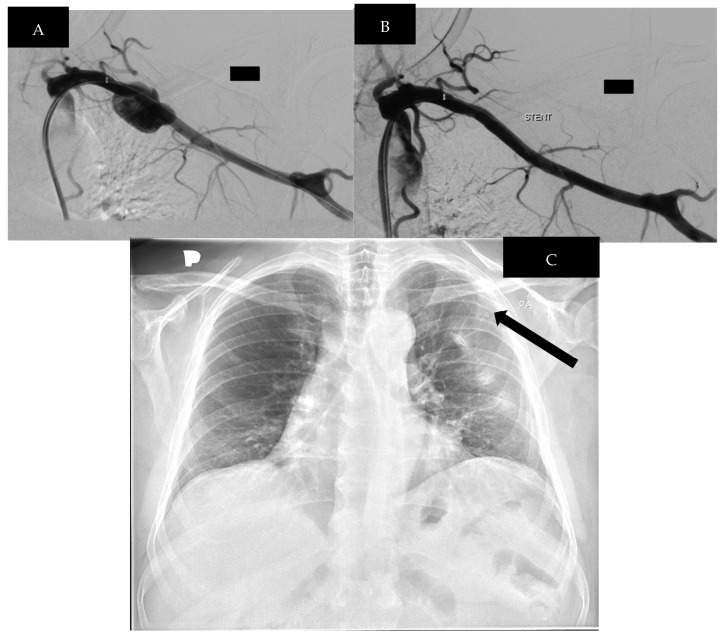
(**A**,**B**) Digital subtraction angiography before and after stent implantation. (**C**) Chest X-ray—showing implanted Viabahn stent. (black arrow).

The patient was immediately transported to the Department of Interventional Radiology, where a left SCA arteriography was performed via a right common femoral artery puncture. At the time of admission, the CRP level measured 1.07 mg/L, and the D-dimer was 603.0 μg/L. A Viabahn covered stent was successfully implanted to restore arterial continuity and exclude the pseudoaneurysm (Figure 6). The puncture site was without signs of hematoma, and the patient was discharged in good general condition.

A follow-up ultrasonography Doppler examination 2 months later confirmed the stent patency. A coagulated pseudoaneurysm about 4 cm long was detected, compressing the subclavian vein at the level of the lesion. Subsequent observations at 2 and 4 months showed no evidence of worsening of the patient’s overall condition. Furthermore, the patient underwent echocardiography, which revealed no abnormalities. Doppler ultrasonography of the left SCA indicated regression of the coagulated pseudoaneurysm, to 20 mm in length. More than a year after the injury, the patient has fully recovered, returned to regular activities, and remains in good health. Scheduled follow-up Doppler ultrasound examinations will continue to monitor the patient’s progress.

## 3. Discussion

Clavicle fractures are relatively common, accounting for approximately 2.6–5% of all fractures. However, associated damage to the SCA as a consequence of these injuries is rare, occurring in approximately 1% [12,13] of patients. Most commonly they are associated with blunt trauma [11]. Our review of medical databases identified only 19 cases described by 14 authors focusing exclusively on post-traumatic SCA pseudoaneurysm patients who received strictly endovascular surgery. Notably, the reports of cases 11 to 16 lacked detailed information regarding the nature of the injuries, only mentioning their post-traumatic origin. A summary of these cases, including their characteristics, is provided in Table 1.

The patient whose case we present here suffered blunt trauma to the left side of the thorax with multiple rib fractures and a clavicle fracture as a result of high-energy trauma due to a bicycle accident. This is a common mechanism of injury to this area [11,13]. The patient developed an SCA pseudoaneurysm alongside a pseudo-joint of the clavicle. Subclavian pseudoaneurysms are associated with significant risks, including rupture and thromboembolic complications, both of which pose life-threatening challenges due to the difficulty in controlling bleeding in this region. As such, prompt diagnosis and treatment are critical.

In the described case, the SCA injury was not detected immediately after the injury. The studies we analyzed confirm this diagnostic difficulty in the absence of obvious signs of vascular damage. In only four cases was the diagnosis made immediately after the trauma [18,19,21]. One extremely rare potential risk factor for subclavian artery damage, apart from its anatomical location, could be an arteriovenous malformation. Damage to such a structure may result in the development of a pseudoaneurysm, so it is crucial to maintain vigilance in patients with these conditions [21]. Another factor that may contribute to the risk of pseudoaneurysm is the presence of diseases that weaken the vascular wall. High body mass index, hypertension, diabetes mellitus, and other conditions leading to atherosclerosis have been identified as independent risk factors for other types of pseudoaneurysms [28,29]. However, there are currently no specific data regarding their impact on the subclavian artery, especially in post-traumatic patients. In the analyzed literature, hypertension was reported in only four cases, while diabetes was noted in one. The majority of authors focused more on trauma-related injuries rather than concomitant comorbidities.

Variations in the anatomy of the SCAs can result in differing symptoms depending on the aneurysm’s location. If the aneurysm is situated proximal to the origin of the vertebral artery, it may lead to subclavian steal syndrome. If the pathology is large, it may cause pressure on adjacent structures such as the trachea or esophagus. This may cause symptoms such as hoarseness, speech disorders, dysphagia, or Horner’s syndrome [30,31,32]. Francis et al. presented another complication, involving compression of the brachial plexus by an organized hematoma, which resulted in plexopathy [16]. Plexopathy is a common complication, as the symptom of hand numbness occurred in five patients [14,16,22,23,24]. Similarly, venous compression involving the jugular or subclavian veins can obstruct blood outflow, causing swelling and thrombosis. Subclavian pseudoaneurysm should always be considered when the patient presents with a symptomatic or asymptomatic pulsatile mass located in the clavicular region [8]. Moreover, a bruit during auscultation of the swelling may be an important diagnostic indicator [16,17,21]. In the presented case, the patient also experienced limb swelling, and subclavicular and brachial vein thrombosis and imaging tests confirmed partial compression of the subclavian vein by the pseudoaneurysm. However, the size of the pseudoaneurysm was not significant enough to cause additional symptoms. Initially, local post-traumatic tissue swelling and hematomas obscured the true cause of the symptoms, delaying the diagnosis.

Almadwahi and Haboob, among others, highlight the fact that an SCA pseudoaneurysm can present as either symptomatic or asymptomatic, with the most common symptom being the appearance of an arterial bump in the subclavian region 2–12 weeks after injury [8]. This symptom was not present in our patient. However, the possibility of post-traumatic vascular complications was highlighted by reduced left upper extremity function and persistent edema.

A chest X-ray is often the first routine examination after chest trauma. However, Doppler ultrasonography should be an obligatory adjunct to the examination in order to assess vascular flow and exclude thrombosis [32]. Such an examination is particularly important because it is cheap, quick, and does not expose the patient to radiation, so it is mandatory as an adjunct to clinical imaging [33]. Ultrasound is a relatively subjective examination and can be inconclusive. More accurate imaging methods, such as angiographic CT or angiographic magnetic resonance imaging, are now readily available and provide greater diagnostic precision. However, Doppler ultrasound is also a key element of post-operative follow-up as an assessment of arterial flow in the peripheral arteries of the affected limb. In our patient, the use of this diagnostic method at each follow-up visit allowed the identification of the pseudoaneurysm. Chest CT should be the method of choice because of its ability to determine the morphology (including wall thickness), the size of the pseudoaneurysm, and its location in relation to other structures. Currently, this method is often combined with arteriography, which involves the injection of contrast into the aortic arch or the SCA itself. This method allows visualization of the aneurysm lumen, its location, and the topography of the adjacent vessels. The combination of arteriography with CT or magnetic resonance imaging allows the precise topography of the aneurysm to be visualized in relation to the surrounding structures and the assessment of blood flow in the examined vessel [31,34]. A contrast-enhanced pseudoaneurysm appears clearly as an oval or circular accumulation of contrast material, and partial thrombosis is indicated by areas of low attenuation within the sac. This method can also help detect hemorrhage [35]. Furthermore, the processed data can be used to obtain three-dimensional images used for surgery planning. Nevertheless, it should be remembered that angiography is an invasive examination and should not be used routinely in all cases. The use of methods such as digital subtraction angiography (DSA) is typically prioritized for patients with clearly identifiable vascular trauma and a low likelihood of concurrent severe injuries. However, in high-energy trauma cases, DSA is often performed after CT imaging to guide potential invasive treatments or when CT findings are inconclusive. In the described case, this method was also employed when the patient required endovascular treatment. Potential complications of arteriography include thrombosis, embolism, hematomas, pseudoaneurysms, and arteriovenous fistulas [36].

Pseudoaneurysms are often resected in an elective surgical procedure [37]. Traditional surgical repair of subclavian pseudoaneurysm using supra- and infraclavicular approaches is effective [8], but the presence of the brachial plexus, pleura, and thoracic duct in this area makes the surgical procedure challenging, and some cases require sternotomy. Thus, due to their lower risk of complications and their superior outcomes, the use of endovascular techniques has recently increased [16,21,24]. In this treatment modality, a stent is deployed within the vessel to ensure proper blood flow and reinforce the compromised section of the vessel wall.

Treatment is required due to the potential for rare but serious complications. Stefanczyk et al. presented a case involving the perforation of a systemic artery pseudoaneurysm into the bronchial tree, a very rare complication that can result in life-threatening hemorrhage [22]. Additionally, pseudoaneurysms can sometimes reach significant sizes. For instance, Ghulam et al. described a 79-year-old patient with a pulsating mass measuring 20 × 20 cm, which was identified as an aneurysm [23]. Such large aneurysms in the SCA are exceptionally rare. Lawson et al. also reported a case of a chronic SCA pseudoaneurysm with a non-union clavicle fracture—a presentation similar to our patient’s. In their case, the aneurysm ruptured during a massage but was successfully treated with stent implantation and internal fixation of the clavicle [26].

Open surgery for SCA pseudoaneurysms presents significant challenges due to the complex anatomy of the area, even for experienced surgeons. As a result, minimally invasive techniques such as stenting or embolization are now preferred whenever technically feasible, as they carry lower risks and yield superior outcomes [11]. The presence of significant structures in this area, such as the brachial plexus, pleural capsule, and left thoracic duct, makes open surgery difficult, and it involves a potentially high risk of damaging adjacent structures. Open surgery may also require sternotomy for control of the proximal SCA, so endovascular approaches are now preferred as less complicating [38]. Open surgery of the SCA—sometimes necessary on the right side—requires access via sternotomy and clavicle transection, and on the left side, an anterolateral thoracotomy or ministernotomy up to 4 bpm and transection of the left clavicle [38]. The minimally invasive method was feasible in our patient. Endovascular treatment is recommended for elderly patients with comorbidities. This approach reduces clinical implications such as the risk of complications and intraoperative burden [36]. Although hybrid procedures combining endovascular techniques with open surgery are sometimes required, this approach was unnecessary in our patient [11]. Compared with open surgery, endovascular procedures are generally associated with significantly fewer complications. Reported complications include stent malposition, endoleak, plexopathy, and limb pain, underscoring the importance of careful post-operative monitoring and appropriate prothrombotic treatment. Notably, our patient experienced no post-operative complications.

Minimally invasive techniques are also possible in the management of subclavian pseudoaneurysms. These include ultrasound-guided compression (USGC), thrombin injection, or percutaneous interventions with adjunctive balloon protection [17,34,39,40]. However, these methods are generally limited to smaller, peripheral pseudoaneurysms that do not cause significant mass effect or limb thrombosis [34]. Ultrasound-guided compression (USGC) is an effective modality but has notable limitations, including the risk of complications such as arterial thrombosis or distal embolization. Additionally, it is not suitable for managing extensive post-traumatic injuries [39]. Thrombin injection, which promotes clot formation by converting fibrinogen to fibrin, shares similar limitations with USGC, particularly for large or complex pseudoaneurysms. Percutaneous thrombin injection with balloon protection can be a valuable option, as it helps control thrombus migration during treatment through the use of a balloon catheter, ensuring targeted pseudoaneurysm occlusion [40]. A novel technique for treating SCA pseudoaneurysms is the use of Angio-Seal (Terumo Interventional Systems). The potential use of Angio-Seal is recommended when stenting is contraindicated due to the proximity of vascular branches or the SCA pseudoaneurysm is resistant to thrombin treatment [11]. This approach minimizes damage to surrounding anatomical structures such as vessels or nerves.

The treatment of clavicle fractures varies with the degree of trauma, displacement of clavicular bone and subsequent deformity, and the presence of vascular and neurologic injuries [41,42]. Most minimally displaced midshaft clavicle fractures can be treated non-surgically [42]. If the fracture is displaced or if it is unstable and risks further nerve or vascular injury, surgical stabilization should be considered. Blunt force trauma to the chest wall with rib fractures may also benefit from surgical stabilization [43]. Athletes desiring the quickest return to sport may also elect surgical stabilization. Plating the clavicle allows accurate reduction of the fracture and maintenance of clavicle length, which may allow improved shoulder function. However, the risks of open surgical reduction and stabilization, including SCA and vein injury, should be considered. In the presented case, open surgery would be have been considered highly dangerous. Disruption of a pseudoaneurysm of the SCA could be difficult to control surgically and life-threatening. In our patient, endovascular treatment was the optimal choice. In such cases, the minimally displaced midshaft clavicle fracture should heal with non-operative management

## 4. Conclusions

Given the high risk of thromboembolic events and the potential for rupture leading to life-threatening hemorrhage, it is necessary to highlight the possibility of subclavian artery pseudoaneurysm occurring in a patient with a clavicle fracture, despite its rarity. Furthermore, the optimal management approach is endovascular repair of the pseudoaneurysm with stent implantation. Post-operative care of the patient is also important and should include observation and anticoagulant therapy to prevent stent occlusion with a thrombus. Treatment of accompanying orthopedic injuries, such as pseudarthrosis, can be performed secondary to the management of the SCA pseudoaneurysm.

## Figures and Tables

**Table 1:** Characteristics of included patients. NR: not reported; L/RUE: left/right upper extremity; EL: endoleak; DM: diabetes mellitus; SCA: subclavian artery; SCVT: subclavian vein thrombosis.

Pat. No	First Author	Sex	Age (Years)	Medical History	Diagnosis from Trauma (Months)	Symptoms	Physical Signs	Location of Lesion	Complications
1	Ashraf [14]	M	74	Blunt chest trauma. Fractures of left clavicle and ribs II to IV	24	Acute LUE pain, numbness in forearm and hand, weakness of hand and cyanosis of fingers	Cyanosis of the left hand and fingers and undetectable brachial, radial, and ulnar pulse. Mild limitation of grip of the left hand	Left	NR
2	Cogburn [15]	F	30	Midshaft fracture of the right clavicle. Vascular complication occurring 6 years after open reduction and internal fixation.	120	Sudden and rapidly developing swelling of the right neck region	Soft and pulsating mass in the right supraclavicular fossa	Right	NR
3	Francis [16]	M	20	Stab wound of the left supraclavicular region	0.5	Painless swelling of the left subclavian region	Pulsating swelling of soft consistency, without tenderness in the left supraclavicular region with a healed trauma wound. Bruit during auscultation of the edema.	Left	EL, increased swelling, acute pain, and paresis of the LUE
4	Akuma [17]	M	8	Stab wound of the right clavicular region	0.5	Progressively increasing swelling of the right neck area.	Non-tender edema. Bruit during auscultation of the edema.	Right	NR
5	Enamorado-Enamorado [18]	M	24	Blunt trauma. Traumatic rupture of the left SCA	0	NR	NR	Left	NR
6	Ismazizi [19]	M	53	Blunt trauma. Fracture of the sternum and right clavicle associated with a tear of the right SCA.	0	NR	NR	Right	NR
7	Renger [20]	F	60	Blunt trauma. Middiaphyseal fracture of the right clavicle with dislocation.	5	The rapid appearance of a painful, white, cold right hand.	Weakening of pulse rate on the radial artery	Right	NR
8	Quinones-Baldrich [21]	M	33	Blunt sports-related trauma of an asymptomatic large congenital arteriovenous malformation in the right shoulder and neck	0	Painful, pulsating mass in the right supraclavicular fossa	Bruit during auscultation of the edema.	Right	NR
9	Stefańczyk [22]	F	10	Fracture of the distal part of the left clavicle	2.5	Massive hemoptysis, dyspnea, pain in the left supraclavicular area, increasing symptoms of LUE ischemia with brachial plexus paralysis	Arterio–bronchial fistula into the bronchial tree and alveoli	Left	NR
10	Ghulam [23]	F	79	Fracture of the left clavicle with a persistent small bulge on the neck, present for 9 years post-trauma.	108	Progression of swelling on the left side of the neck, pain in the medial fingers, numbness in the left hand	Pulsatile mass on the left side of the neck	Left	NR
11	Wang [24]	M	51	NR	NR	Chest pain	Pulsatile mass	Right	NR
12		M	59	Hypertension, DM	NR	Chest tightness with hoarseness	NR	Right	NR
13		M	65	Hypertension	NR	Dysphagia	NR	Right	NR
14		M	49	Hypertension	NR	Left shoulder and left chest back pain	NR	Left	NR
15		M	42	NR	NR	Numbness, pain, and weakness of the RUE	Pulsatile mass	Right	NR
16		F	45	NR	NR	NR	Pulsatile mass	Left	NR
17	Perri [25]	M	67	Fracture of the middle clavicle, scapular body, coracoid process, and ribs IV, V, and VI on the left side.	0	Left subclavian pain	Pulsatile mass	Left	NR
18	Lawson [26]	F	30	Midshaft fracture of the left clavicle.	NR	Intermittent left shoulder girdle discomfort and neuropathic symptoms	Focal soft tissue mass in the left supraclavicular fossa	Left	NR
19	Biz [27]	M	88	Midshaft fracture of the right clavicle.	1.5	NR	Soft non-pulsatile mass in the right supraclavicular fossa, bone discontinuity along the clavicle midshaft with abnormal mobility of fracture fragments	Right	NR
20	Presented case	M	49	Fracture of the clavicle and ribs II to VII on the left side, left SCVT, hypertension.	4	Swelling in the left supraclavicular area, left-sided chest pain, subclavicular and brachial vein thrombosis	Upper limbs symmetrical, without swelling, with palpable pulse on the radial arteries	Left	NR

## Data Availability

The data presented in this study are available in this article.
